# Musculoskeletal modelling and simulation of oil palm fresh fruit bunch harvesting

**DOI:** 10.1038/s41598-022-12088-6

**Published:** 2022-05-14

**Authors:** Yon Sin Chan, Yu Xuan Teo, Darwin Gouwanda, Surya Girinatha Nurzaman, Alpha Agape Gopalai, Subbiah Thannirmalai

**Affiliations:** 1 School of Engineering, Monash University Malaysia, Bandar Sunway, 47500, Selangor Malaysia; 2 Monash Industry Palm Oil Research Platform, Monash University Malaysia, Bandar Sunway, 47500 Selangor Malaysia; 3 Sime Darby Technology Centre, Serdang, 43400, Selangor Malaysia

**Keywords:** Biomedical engineering, Health occupations

## Abstract

Oil palm harvesting is a labor-intensive activity and yet it was rarely investigated. Studies showed that complementing human motion analysis with musculoskeletal modelling and simulation can provide valuable information about the dynamics of the joints and muscles. Therefore, this study aims to be the first to create and evaluate an upper extremity musculoskeletal model of the oil palm harvesting motion and to assess the associated Musculoskeletal Disorder (MSD) risk. Tests were conducted at a Malaysia oil palm plantation. Six Inertial Measurement Units (IMU) and Surface Electromyography (sEMG) were used to collect kinematics of the back, shoulder and elbow joints and to measure the muscle activations of longissimus, multifidus, biceps and triceps. The simulated joint angles and muscle activations were validated against the commercial motion capture tool and sEMG, respectively. The muscle forces, joint moments and activations of rectus abdominis, iliocostalis, external oblique, internal oblique and latissimus dorsi were investigated. Findings showed that the longissimus, iliocostalis and rectus abdominis were the primary muscles relied on during harvesting. The harvesters were exposed to a higher risk of MSD while performing back flexion and back rotation. These findings provide insights into the dynamical behavior of the upper extremity muscles and joints that can potentially be used to derive ways to improve the ergonomics of oil palm harvesting, minimize the MSD risk and to design and develop assistive engineering and technological devices or tools for this activity.

## Introduction

Investigating human motion is important in many fields for different purposes, such as improving worker's efficiency, posture and working condition. A comprehensive human motion analysis can provide essential biomechanical characteristics of the joint and muscle^1^. Motion capture technologies such as the optical Motion Capture System (MoCap), Inertial Measurement Unit (IMU) and Electromyography (EMG), are commonly used to quantify human motion^2^. Although these technologies can provide an overall outlook of the motion, they have limited capabilities. MoCap and IMU can only determine joint kinematics whereas the EMG can only measure muscle activations. Using them together synchronously can be laborious and time consuming—making them impractical for field study. Musculoskeletal modelling and simulation can overcome this limitation. It can determine the joint kinematics and muscle activations using basic dynamical properties of the motion. It is less invasive and can provide important biomechanical information that is impractical to obtain directly.

OpenSim (SimTK, Stanford, CA, USA) and Anybody Modeling System (Anybody, Aalborg, Denmark) are some of the popular tools used to perform musculoskeletal modelling and simulation in various activities such as walking^3^, running^4^, lifting tasks^5^ and bowling (cricket)^6^. Both software allow the user to create a musculoskeletal model, either building it from scratch or modifying the existing one. The users can also perform relevant computation, e.g., kinematic analysis, inverse dynamics analysis and static optimization^7^. However, earlier versions of OpenSim only allow the input kinematics data from MoCap. A newer version of OpenSim 4.1, released in 2020, added a new feature to process the IMU kinematics data. This feature is important because IMU is small and light, easy to set up and can produce comparable results with MoCap^8^. It also allows the motion to be captured outside the laboratory environment. These attributes, in turn, produce a more realistic data that accurately represent the measured activity. As a result, IMU has been used in various applications including walking, sitting, climbing stairs, lifting, pushing and pulling activities^9^.

One of the fields where human motion analysis can be beneficial is agriculture. Palm oil has been the fastest-growing vegetable oil globally since 2004^10^. Despite the increasing global demand of palm oil, most plantation activities are performed manually by workers, including the Fresh Fruit Bunch (FFB) harvesting. During FFB harvesting, the harvesters need to visually identify and harvest the ripe FFB with a large force repeatedly in an awkward posture^11^, exposing them to a higher risk of Musculoskeletal Disorder (MSD)^12–16^. Qualitative approaches such as questionnaires^12,13^, interviews^12,14^, Rapid Entire Body Assessment (REBA)^15^ and Rapid Upper Limb Assessment (RULA)^16^ were employed to investigate this activity. A recent study reported in^11^ quantitatively examine the FFB harvesting using IMU and EMG. Although the existing studies sufficiently pointed out that the harvesters are facing MSD risk, most biomechanical parameters of the harvesters remain unclear. The lack of detailed quantitative assessment on harvester motion forms a barricade to strategies that can improve the ergonomics of the harvesting motion and mitigate the MSD risk.

A computational model that can represent the harvesting motion has the potential to (1) elucidate the underlying biomechanical characteristics, (2) investigate the MSD risk associated with FFB harvesting and (3) serve as the fundamentals to improve the posture and mitigate or reduce the MSD risk. To date, no FFB harvesting musculoskeletal model has been developed and validated. Therefore, this study aims to create an upper extremity musculoskeletal model that can represent the FFB harvesting motion and can be used to investigate the kinematics behavior of the back, shoulder and elbow, and their muscle activations. To validate this model, the joint angles of the back, shoulder and elbow were investigated and compared with results estimated by the commercial motion capture software. The muscle activations of the longissimus, multifidus, biceps and triceps were evaluated against the measurements collected using surface Electromyography (sEMG). Lastly, this study investigates the quality of the posture using the method proposed in^17^ and evaluates the MSD risk.

## Methodology

### Musculoskeletal model and simulation

A full-body musculoskeletal model created by Erica et al*.*^18^ was modified to produce a model that is suitable for FFB harvesting activity. The original model has 30 body segments, 238 musculotendon actuators, 29 degrees of freedom and eight upper extremity Hill-type muscle groups, including the multifidus, rectus abdominis, psoas major, internal obliques, external obliques, quadratus lumborum, latissimus dorsi and erector spinae (longissimus and iliocostalis) muscles. The thoracic and cervical parts of the spine, ribcage, head and scapula were modelled as a rigid body, called the torso, with spherical joints between six intervertebral joints (T12-L1, L1-L2, L2-L3, L3-L4, L4-L5, L5-S1). The vertebrae followed a spinal rhythm that distributes the trunk motion over these six intervertebral joints through linear kinematic coordinate coupling constraints^18^. Three main modifications were made to the model as listed below:

1. The original Range of Motion (ROM) of shoulder and elbow joints were much greater than the Normative Range of Motion (NROM); hence they were altered to the NROM^19^, as shown in Table 1. This modification prevents an awkward posture from being generated in OpenSim.
2. Hill-type biceps and triceps muscles were added. The muscle architectural parameters were adopted from an existing upper-extremity OpenSim model by *Daniel *et al*.*^20^, used in pulling and pushing tasks. The Optical Muscle Fibre Length (OMFL), Tendon Slack Length (TSL) and Pennation Angle (PA) of biceps and triceps were sourced from a cadaver study of^21^. The TSL of biceps was modified to preserve its operating ranges at the proximal joint it crosses^22^. The Peak Force (PF) was obtained based on the measured muscle volumes of^23^ and isometric strength of^24^. Overall, these parameters (OMFL, PF, TSL, PA) were based on the data of young male adults from different literatures^21,23,24^ and were reported as follows:
3. OMFL: 11.6 cm (biceps long), 13.2 cm (biceps short), 13.4 cm (triceps long), 11.4 cm (triceps lateral), 11.4 cm (triceps medial)
4. PF: 525.1 N (biceps long), 316.8 N (biceps short), 771.8 N (triceps long), 717.5 N (triceps lateral), 717.5 N (triceps medial)
5. TSL: 27.8 cm (biceps long), 20.0 cm (biceps short), 14.3 cm (triceps long), 9.8 cm (triceps lateral), 9.1 cm (triceps medial)
6. PA: 0° (biceps long), 0° (biceps short), 12° (triceps long), 9° (triceps lateral), 9° (triceps medial)^22^
7. Reserve actuators were added to the back joint (L5-S1), shoulder joints and elbow joints to provide extra actuation when the muscles cannot generate sufficient accelerations at a certain time. The optimal force of the reserve actuator (F_RA_) must be low enough to ensure that the muscles are the main contributor to the net joint moments (i.e., the joint moment produced by the summed or net effect of all the structures)^1,25^. However, there is no recommended F_RA_ for harvesting activity. Hence, five different F_RA_ values: 10 N, 20 N, 30 N, 40 N and 50 N were investigated. They were used to perform the inverse dynamics and calculate the net joint moments. Static optimization was performed to determine the reserve actuator moments. The peak reserve actuator moments at different F_RA_ were normalized to the peak net joint moments. The results are presented in Table 2. The peak residual forces (F_X_, F_Y_, F_Z_) and peak residual moments (M_X_, M_Y_, M_Z_) were added to the table. Since the normalized peak reserve actuator moments has to be less than 10%^26^, only the F_RA_ of 30 N, 40 N and 50 N were selected to estimate the muscle activation.

**Table 1:** ROM of the back, shoulder and elbow of the Erica et al.'s model and the proposed model (*FE* Flexion–Extension, *LB* Lateral Bending, *AA* Adduction-Abduction).

Joint Motions	*Erica *et al*.*'s model^18^ joint angle (Degree)		The proposed model joint angle (Degree)	
	Minimum	Maximum	Minimum	Maximum
Back FE	− 80 (Flexion)	26 (Extension)	− 80 (Flexion)	26 (Extension)
Back LB	− 25 (Left)	25 (Right)	− 25 (Left)	25 (Right)
Back Rotation	− 56 (Left)	56 (Right)	− 56 (Left)	56 (Right)
Shoulder FE	− 90 (Extension)	180 (Flexion)	− 50 (Extension)	180 (Flexion)
Shoulder AA	− 180 (Abduction)	90 (Adduction)	− 180 (Abduction)	0 (Adduction)
Shoulder Rotation	− 140 (Internal rotation)	100 (External rotation)	− 80 (Internal rotation)	60 (External rotation)
Elbow FE	− 10 (Extension)	160 (Flexion)	0 (Extension)	140 (Flexion)

**Table 2:** Average normalized peak reserve actuator moments of different joint motions (*n* = 6), peak residual forces and peak residual moments at five different F_RA_.

F_RA_	10 N	20 N	30 N	40 N	50 N
**Average normalized peak reserve actuator moments of different joint motions (%)**					
Back FE	26.83	13.32	8.80	6.61	5.18
Back LB	8.46	4.24	2.83	2.32	1.73
Back Rotation	18.05	8.93	5.56	5.22	3.00
Shoulder FE	6.49	3.34	2.24	1.42	1.36
Shoulder AA	6.71	3.69	2.50	3.29	1.51
Shoulder Rotation	7.95	4.02	2.62	2.34	1.54
Elbow FE	9.05	4.53	3.02	1.70	1.81
**Peak residual forces (N)**					
F_X_	134.48	67.24	44.83	44.68	26.90
F_Y_	127.48	63.74	42.49	32.60	25.50
F_Z_	144.01	72.01	48.00	16.83	28.80
**Peak residual moments (Nm)**					
M_X_	112.69	56.35	37.56	22.12	22.54
M_Y_	29.06	14.53	9.69	7.32	5.81
M_Z_	100.43	50.62	34.03	31.82	20.40

Two assumptions were made in this study. The first assumption is that the exerted harvesting force of 303.5 N is the sum of the pulling force produced by the harvesters (300 N^27^) and the weight of the harvesting tool (3.5 N^28^). The second assumption is that the harvesting force was equally distributed to the left and right hands. Hence, an external force of 151.75 N was added to each hand. The exerted harvesting force was found in^27^, which investigated the force and energy required to perform the harvesting motion with a test jig. The authors then performed a functional test on their prototype magnetic oil palm cutter at an oil palm plantation and the harvesting force was found to be reasonable to harvest the fruit^27^. The weight of the harvesting tool was obtained in^28^ because it is the harvesting tool used by the harvesters in this study.

Inverse kinematics was performed to calculate the joint angle using orientations measured by the IMU. This joint angle is denoted as *θ*_*OS*_. Static optimization was then used to estimate the muscle activation. It resolves the net joint moment into individual muscle force at each instant while minimizing the sum of squared muscle activation^29^. It is the most conventional approach to calculate muscle activation in a dynamic activity due to its robustness, higher efficiency and independence from the experimental EMG^30^. The simulated activations of all the muscle fascicles for each muscle were added together^18^. For example, the estimated muscle activation of the longissimus is the total activations of the lumbar and thoracic components of the longissimus thoracis. The same applies to the multifidus, biceps and triceps.

### Experiment

The experiment was conducted at a Malaysia oil palm plantation. It involved six experienced right-handed male oil palm FFB harvesters (33.5 ± 6.0 years old, 168.83 ± 4.74 cm, 56.83 ± 4.26 kg). They provided written consent after they were briefed on the experiment objectives and procedures. Informed written consent for publication was obtained. This study was reviewed and approved by Monash University Human Research Ethics Committee (MUHREC).

**Figure 1:**
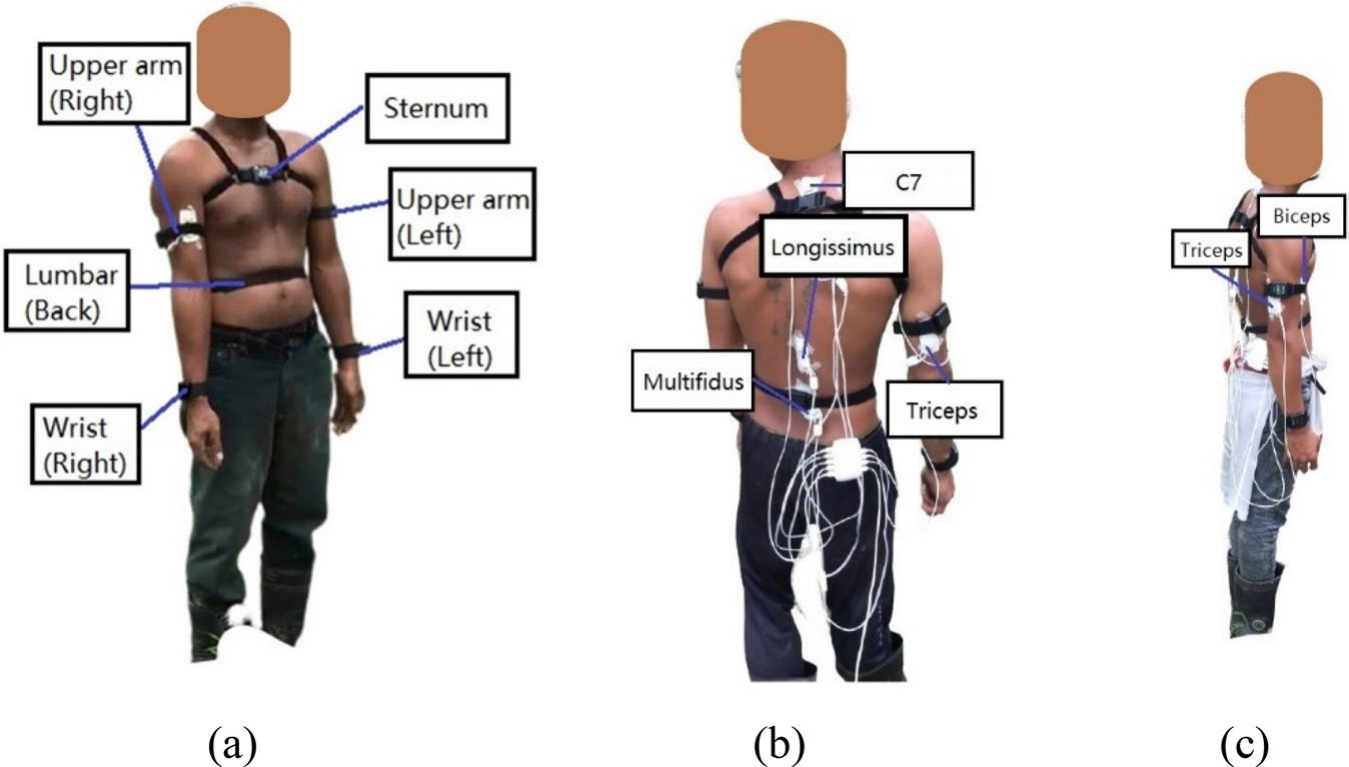
Location of (**a**) IMU sensors on the harvester, (**b**) EMG electrodes on muscles of interest from posterior view and (**c**) right side view, with reference electrode at the C7 region.

Six IMU sensors (APDM OPAL, Portland, OR, USA) were placed on the harvesters' sternum, lumbar, upper arms and wrists, as shown in Fig. 1a. EMG electrodes (Biosignalplux, Lisbon, Portugal) were placed on the right side of the upper extremities to measure the muscle activations of the biceps, triceps, multifidus and longissimus with reference electrode at the C7 region (Fig. 1b and c). The locations of IMU sensors and EMG electrodes were based on the recommendations of the sensor proprietary software, Moveo Explorer^31^ and SENIAM convention^32^, respectively. Both sensors were synchronized using an external trigger. The sampling frequency of IMU and EMG sensors were 128 Hz and 1000 Hz, respectively. The harvesters were requested to stand upright and remain stationary for three seconds for sensor calibration. They then performed the harvesting activity for one minute. The harvesters only harvested FFB from trees with a height between 3 and 5 m for consistency purposes. A video camera was used to record the harvesting activity.

The IMU data were filtered with a 4th order Butterworth low-pass filter with a cut-off frequency of 6 Hz. The EMG data were processed using codes written in MATLAB (Mathworks, Nantucket, MA, USA). The data were filtered with a band-pass filter with 20 Hz and 450 Hz cut-off frequencies. They were then full-wave rectified and smoothened using a moving-average filter with a window size of 1000 ms^33^. Peak Dynamic Method was applied to normalize the EMG data of each muscle to the maximum muscle activation of each harvester. This method replaces the conventional Maximum Voluntary Contraction (MVC) normalization method^34^ because of the remoteness of the plantation sites, which made it challenging to acquire the muscle MVC.

**Figure 2:**
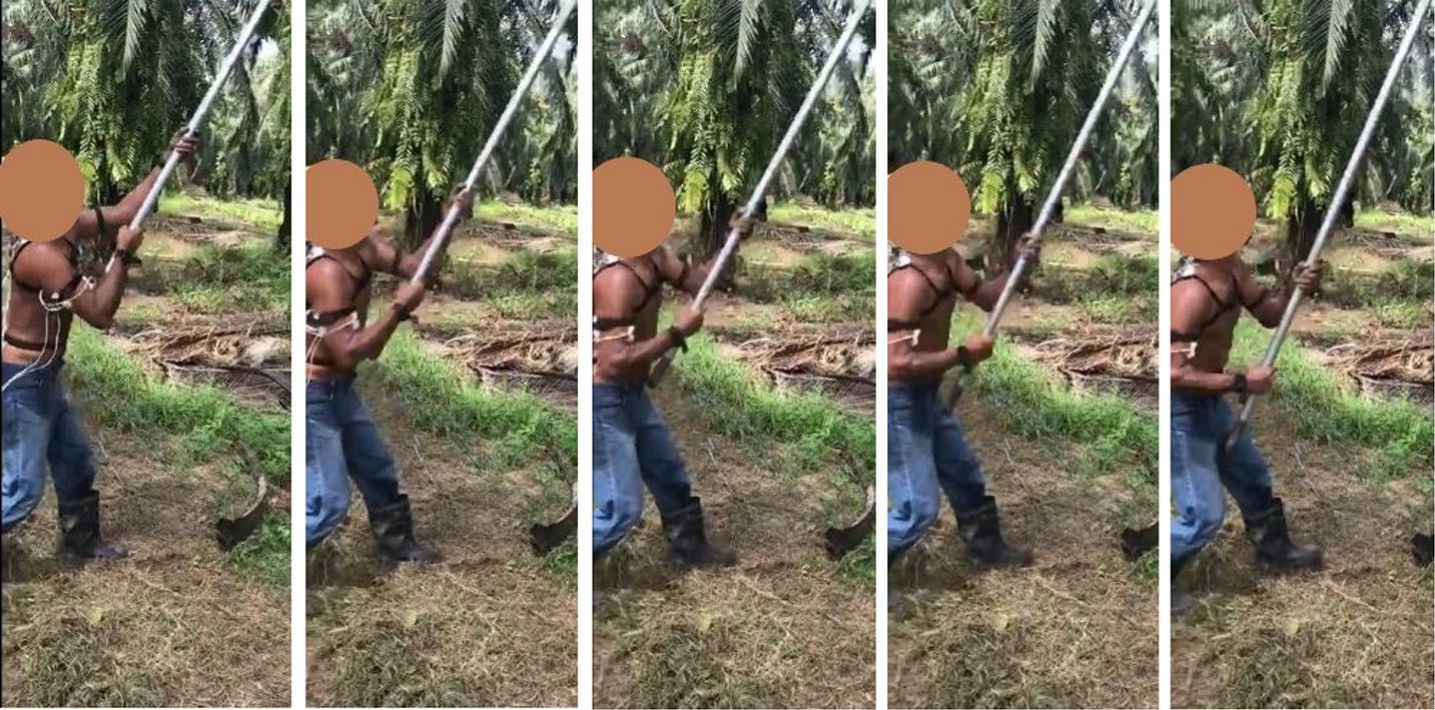
A sequence of the harvesting motion.

From the recorded video footage, it can be observed that the harvesting process is complex. The harvester needs to move around the tree, identify the ripe FFB and cut the fronds, that might interrupt the FFB harvesting. The harvester then positions the sickle on the FFB stalk and pull the sickle downward to cut the stalk, as shown in Fig. 2. For a more accurate and representative analysis, the IMU and EMG data for each harvesting motion of different time lengths were extracted, linearly interpolated and averaged to produce a harvesting motion with the same timeframe^35^.

### Model validation

The joint angle *θ*_*os*_ was compared and validated with the joint angle obtained from the IMU proprietary software, Moveo Explorer. The Pearson Correlation Coefficient (R) was calculated to determine the strength of association between them. The closer the R to zero, the weaker the strength of association. Root Mean Square Error (RMSE) was also calculated to identify the error between them. A smaller RMSE value indicates a smaller difference between them. However, there is no clear guideline on the acceptable R and RMSE values. Hence, they were compared with the recent work^36^, which covers the range of R and RMSE that show good to excellent validity.

For comparison purposes, the muscle activations measured by EMG were resampled to 128 Hz to match the IMU sampling rate. They were then normalized to the maximum muscle activation of each harvester. The simulated normalized muscle activations were then compared and validated with experimental data. The R and Mean Absolute Error (MAE) were calculated and compared with studies^37,38^, that categorize the range of R and MAE into different qualities of correlation, shown in Table 3.

**Table 3:** Different qualities of correlation of the muscle activation^37,38^.

	Value	Quality of correlation
R	0.9–1.0	Very high
	0.7–0.9	High
	0.5–0.7	Moderate
	0.3–0.5	Low
	0.0–0.3	Negligible
MAE	< 0.10	Excellent
	0.10–0.20	Good
	> 0.20	Poor

### Quality of harvesting posture

Other than the joint angle and muscle activation, the harvesting model was also used to compute the joint moment, muscle force and muscle activation of the other muscles that were not measured during the experiment i.e., rectus abdominis, iliocostalis, external oblique, internal oblique and latissimus dorsi. The peak joint moments were normalized to each harvester’s body weight, averaged and tabulated. Higher joint moment indicates that the harvester faces a higher risk of MSD when performing that joint motion, and vice versa. To identify the active muscle used during harvesting, the normalized peak muscle activations and the normalized peak muscle forces at different F_RA_ of 30 N, 40 N and 50 N were calculated, expressed in percentage and then ranked accordingly.

The maximum ROM of each joint was identified to determine the potential stressful joint motion. The Discomfort Value (DV)^17^ was used as a measure to identify the quality of the harvesting posture. It uses the ROM of the joint to determine the DV and then classifies it into different levels of perceived discomfort that correspond to the good, so-so and poor postures. Different joint motions have different DV. The relationship between NROM, DV and quality of posture for the back flexion is shown in Table 4. For example, if the ROM of the harvester's back flexion is 39° and the NROM of this motion is 80°^19^, the harvester's back flexion will be equal to 48.75% of the NROM and will have a DV value of 22.08, which is associated with a so-so posture. A higher DV value corresponds to a higher risk of MSD and indicates that the joint experiences greater stress. These results were evaluated together with the simulated muscle activation, muscle force and joint moment to identify the muscle or joint that has a higher risk of MSD.

**Table 4:** A relationship between the back flexion NROM, DV and quality of postures^17,19^.

NROM	DV	Quality of postures
0%	< 22.08	Good
25–75%	22.08–65.13	So-so
100%	> 65.13	Poor

## Result and analysis

### Simulation results

**Figure 3:**
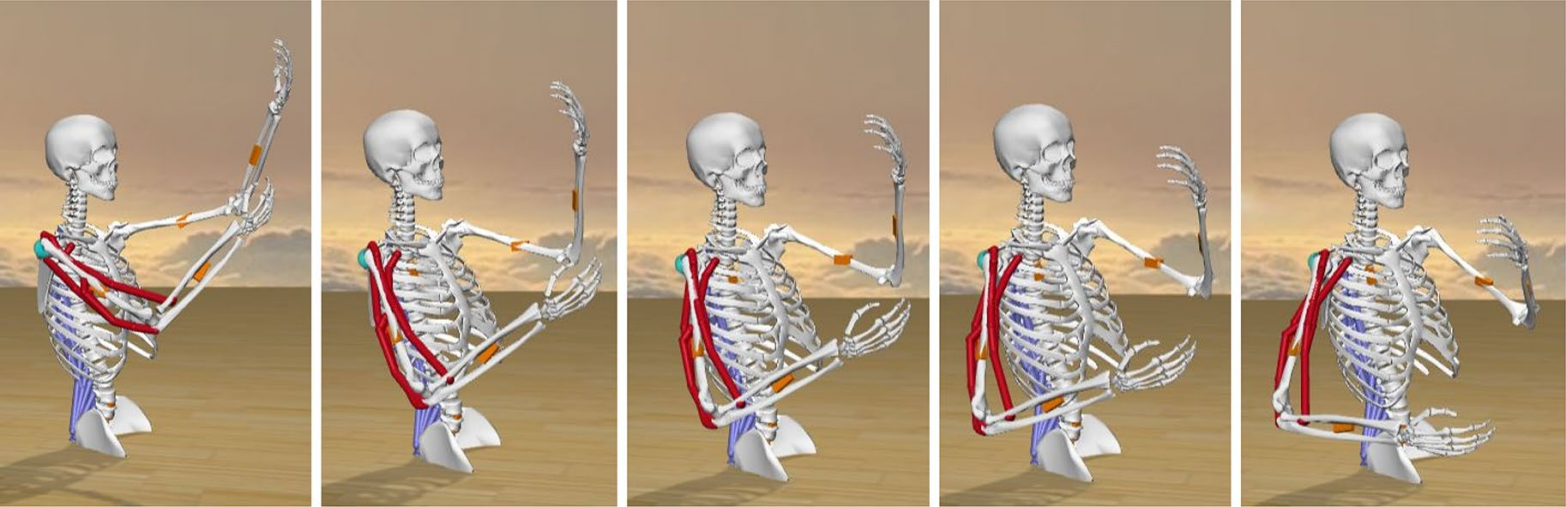
A sequence of the simulated harvesting motion of a harvester.

A sequence of the simulated harvesting motion is shown in Fig. 3. It can be observed that the following motions were performed during harvesting: back flexion, back lateral bending to the right, back rotation to the right, shoulder extension (left, right), shoulder adduction (left), shoulder internal rotation (left), shoulder abduction (right), shoulder external rotation (right), elbow flexion (left) and elbow extension (right). It took approximately 0.3 s to execute this movement. The extremely short duration of harvesting motion suggests that the harvesters exerted a very large force to cut the stalk to harvest the fruit. If this motion is performed repetitively, it exposes them to the greater risk of MSD.

Table 5 shows the average R and RMSE of the back, shoulder and elbow joint angles (R_θ_, RMSE_θ_). These values correlate well with the good to excellent validity discussed in^36^. The R_θ_ and RMSE_θ_ of all the joint motions were well within the acceptable range. These results serve as solid evidence that the proposed musculoskeletal model can produce comparable results with the commercial IMU-based motion capture software.

**Table 5:** The average R and RMSE of the back, shoulder and elbow joint angles (R_θ_, RMSE_θ_) and the good to excellent R_θ_ and RMSE_θ_ in^36^ (*FE* Flexion–Extension, *LB* Lateral Bending, *AA* Adduction-Abduction).

Motion	R_θ_	Good to excellent R_θ_ in^36^	RMSE_θ_	Good to excellent RMSE_θ_ in^36^
**Back**				
FE	0.98	0.72–0.99	2.64°	1.80°–5.90°
LB	0.85	0.72–0.99	2.46°	1.80°–5.90°
Rotation	0.95	0.72–0.99	1.84°	1.80°–5.90°
**Shoulder (dominant side)**				
FE	0.99	0.69–1.00	9.94°	≤ 15.00°
AA	0.88	0.69–1.00	8.15°	< 20.00°
Rotation	0.90	0.69–1.00	11.11°	1.00°–60.00°
**Shoulder (non-dominant side)**				
FE	1.00	0.69–1.00	11.51°	≤ 15.00°
AA	0.81	0.69–1.00	13.76°	< 20.00°
Rotation	0.84	0.69–1.00	14.15°	1.00°–60.00°
**Elbow (dominant side)**				
FE	0.86	0.85–0.99	11.87°	0.20°–30.60°
**Elbow (non-dominant side)**				
FE	0.99	0.85–0.99	20.22°	0.20°–30.60°

Table 6 shows the average R and MAE (R_MA_, MAE_MA_) of the muscle activation of the longissimus, multifidus, biceps and triceps at different F_RA_. Longissimus and triceps showed high correlations of R_MA_ and good correlations of MAE_MA_ at all F_RA_. Multifidus showed moderate (F_RA_ = 40 N) to high (F_RA_ = 30 N, 50 N) correlations of R_MA_ and good correlation of MAE_MA_ at all F_RA_. Biceps showed moderate (F_RA_ = 40 N, 50 N) to high (F_RA_ = 30 N) correlations of R_MA_ and poor correlation of MAE_MA_ at all F_RA_. Overall, the simulated activations of the longissimus and triceps showed good correlations with the EMG data at all F_RA_. On the other hand, biceps showed the best correlation when the F_RA_ was 30 N, whereas multifidus showed the worst correlation when the F_RA_ was 40 N.

**Table 6:** The average R and MAE of the muscle activations for the longissimus, multifidus, biceps and triceps between OpenSim and EMG (R_MA_, MAE_MA_) at different F_RA_.

Muscle	R_MA_ at different F_RA_		
	30 N	40 N	50 N
Longissimus	0.86	0.84	0.87
Multifidus	0.87	0.67	0.87
Biceps	0.72	0.68	0.66
Triceps	0.72	0.77	0.78

Muscle	MAE_MA_ at different F_RA_		
	30 N	40 N	50 N
Longissimus	0.10	0.13	0.10
Multifidus	0.13	0.13	0.14
Biceps	0.24	0.24	0.23
Triceps	0.13	0.18	0.18

Generally, it is more difficult to obtain a good correlation in the muscle activation between simulation results and experimental data compared to joint angle. It is because many parameters may affect muscle activation in a simulated environment. These parameters include the estimated joint angle, harvesting force, muscle architectural parameter and reserve actuator. Moreover, the limitations of sEMG, such as movement artefact, also may contribute to the discrepancies. On the other hand, only the limitations of IMU and the length of the body segment may affect the estimated joint angle. Hence, the recommended values of R to represent good validity are generally lower for muscle activation than for joint angle.

**Figure 4:**
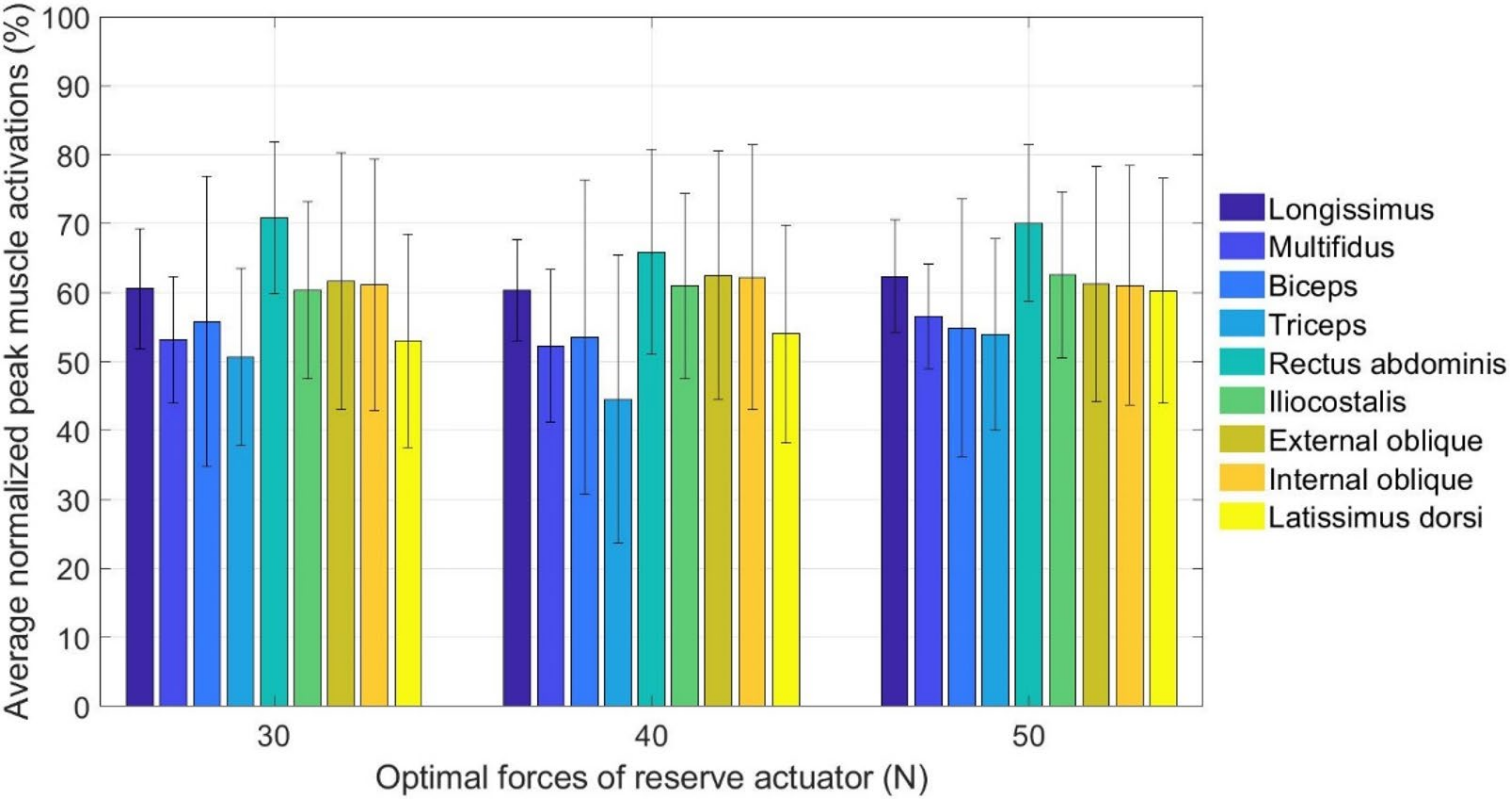
Average normalized peak muscle activations of harvesters at different F_RA_ – 30 N, 40 N and 50 N.

Figure 4 shows the average normalized peak muscle activation of the harvesters when F_RA_ were 30 N, 40 N and 50 N. Higher activation value indicated that the muscle was more active than the others during harvesting. Despite the small differences in the rankings, some similar patterns can be observed. The rectus abdominis was the most active muscle, whereas the triceps was the least active muscle. The external oblique, internal oblique, longissimus and iliocostalis were more actively used than multifidus when performing the back motions. These findings revealed that rectus abdominis was actively used during harvesting, followed by internal oblique, external oblique, longissimus and iliocostalis.

**Figure 5:**
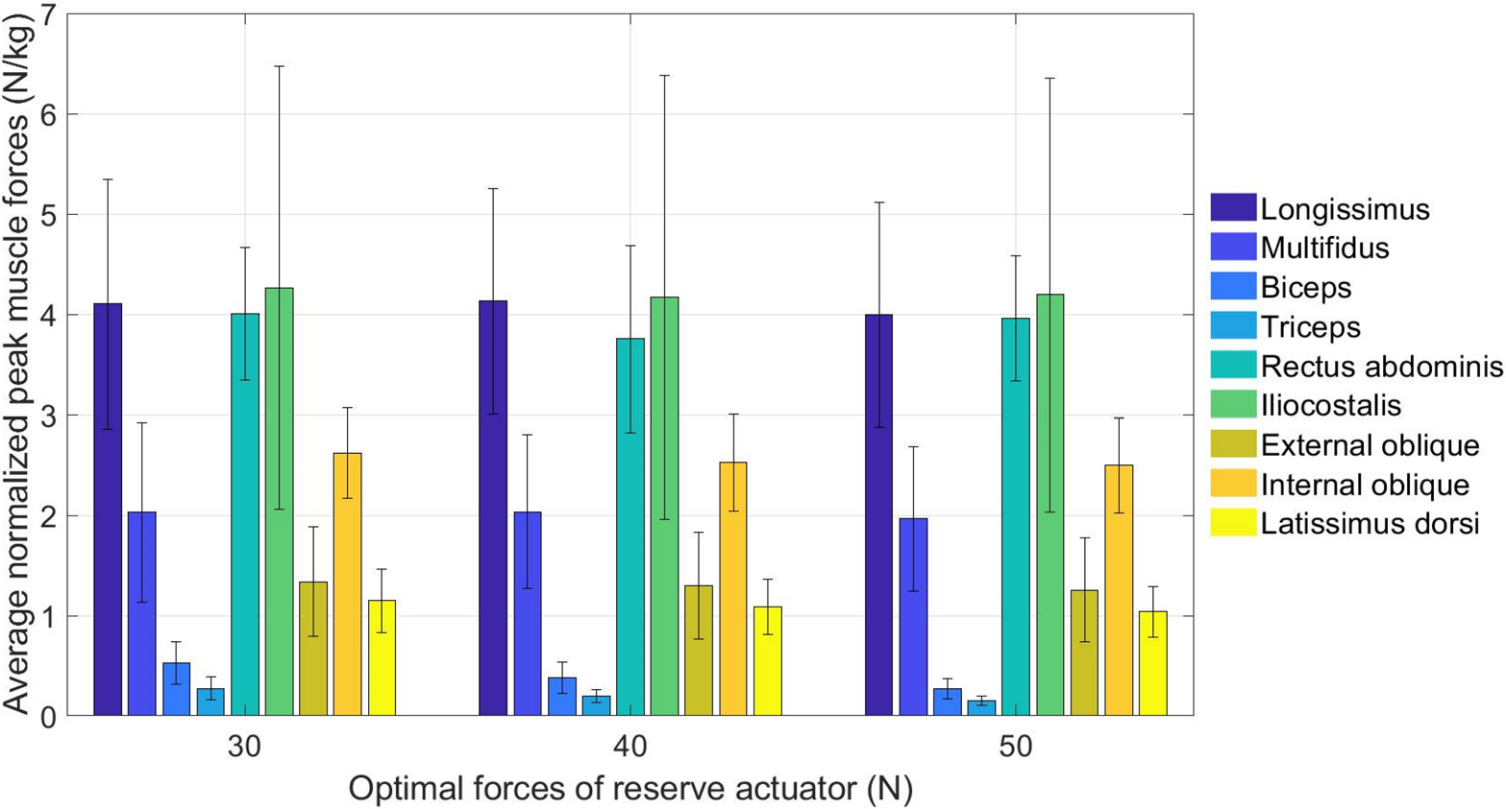
Average normalized peak muscle forces of the harvesters at different F_RA_ – 30 N, 40 N and 50 N.

Figure 5 shows the average normalized peak muscle force when F_RA_ were 30 N, 40 N and 50 N. Similar ranking was found at all F_RA_, as follows: Iliocostalis, longissimus, rectus abdominis, internal oblique, multifidus, external oblique, latissimus dorsi, biceps, triceps. The iliocostalis, longissimus and rectus abdominis showed significant muscle forces, indicating a higher risk of MSD on these muscles.

Table 7 shows the average peak joint moment during harvesting. The back lateral bending produced the greatest joint moment, followed by the back flexion, dominant shoulder extension and back rotation. These four joint motions displayed notable joint moments, suggesting a higher risk of MSD when performing these movements.

**Table 7:** Average peak joint moment during harvesting.

Motion	Average peak joint moment (Nm/kg)
**Back**	
Flexion	13.69
Lateral bending to the right	19.02
Rotation to the right	5.23
**Shoulder (dominant)**	
Extension	5.98
Abduction	1.46
Rotation	0.58
**Shoulder (non-dominant)**	
Extension	0.79
Adduction	1.17
Rotation	1.81
**Elbow (dominant)**	
Elbow extension	0.79
**Elbow (non-dominant)**	
Elbow extension	0.37

### Harvesting posture quality and potential risk of MSD

Table 8 shows the percentage of harvesters with poor, so-so and good postures for different types of back, shoulder and elbow joint motions. The muscles responsible for each joint motion were included. Some or all harvesters showed so-so postures at the back flexion (33.33%), back rotation (100.00%), dominant shoulder rotation (33.33%), non-dominant shoulder flexion (66.67%), non-dominant shoulder rotation (33.33%) and both elbow flexions (100%). No poor posture was observed during harvesting. The joint motion with so-so posture indicates the potential of stressful joint motion, indicating the risk of MSD.

**Table 8:** Percentage of harvesters with poor, so-so and good postures for different types of back, shoulder and elbow joint motions (*n* = 6)and their associated muscles^39^.

Motion	Muscle	Poor posture (%)	So-so posture (%)	Good posture (%)
**Back**				
Flexion	Rectus abdominis	0.00	33.33	66.67
LB	Longissimus, multifidus, iliocostalis, internal oblique, external oblique	0.00	0.00	100.00
Rotation		0.00	100.00	0.00
**Shoulder (dominant side)**				
Flexion	Biceps	0.00	0.00	100.00
Adduction	Triceps, latissimus dorsi	0.00	0.00	100.00
Rotation	Infraspinatus, teres minor, teres major, latissimus dorsi	0.00	33.33	66.67
**Shoulder (non-dominant side)**				
Flexion	Biceps	0.00	66.67	33.33
Adduction	Triceps, latissimus dorsi	0.00	0.00	100.00
Rotation	Infraspinatus, teres minor, teres major, latissimus dorsi	0.00	33.33	66.67
**Elbow (dominant side)**				
Flexion	Biceps	0.00	100.00	0.00
**Elbow (non-dominant side)**				
Flexion	Biceps	0.00	100.00	0.00

**Figure 6:**
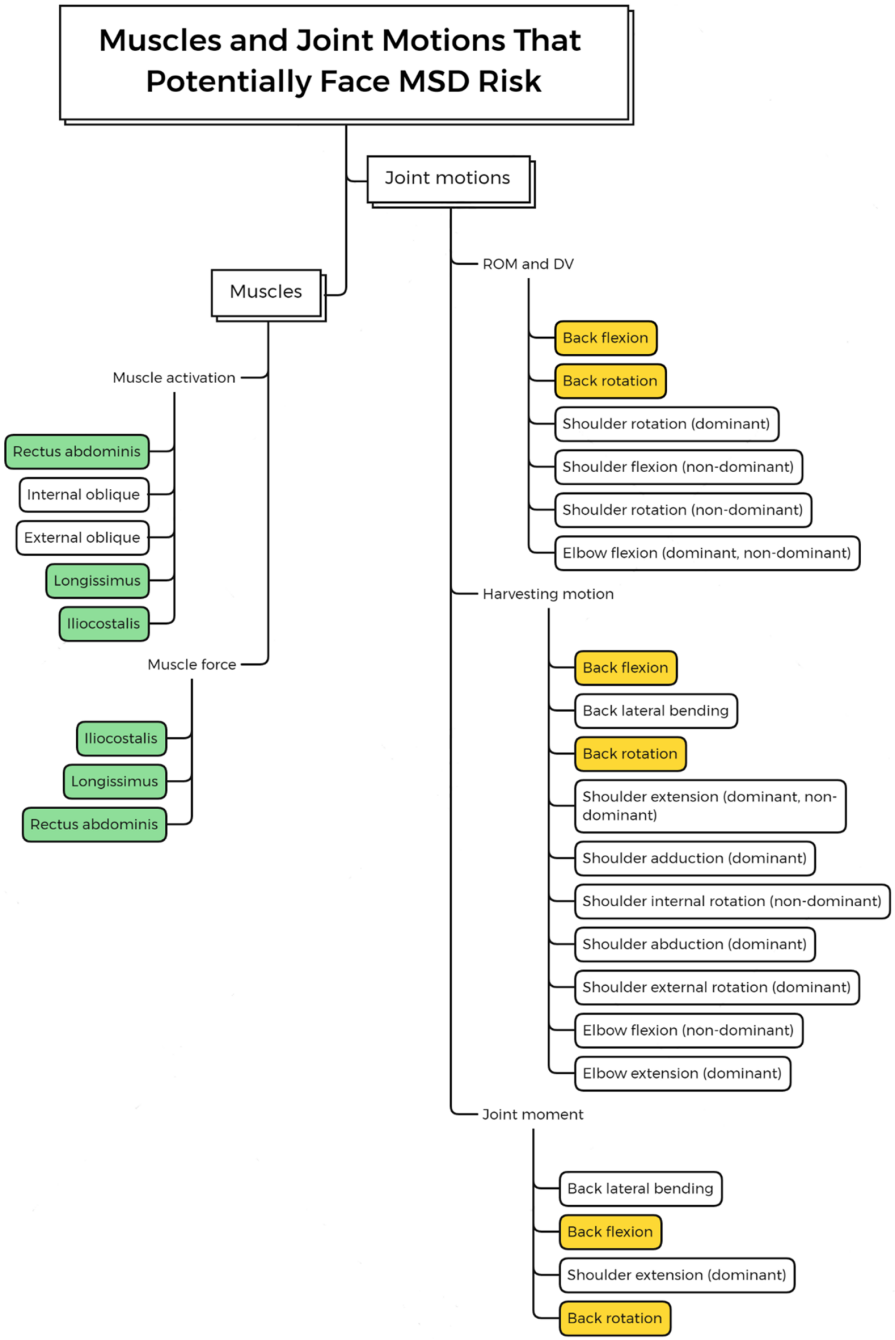
Muscles and joint motions that potentially face MSD risk.

The joint angle, joint moment, muscle activation and muscle force provide essential information about the potential MSD risk of the harvester from different perspectives. To fully understand and identify the MSD risk during harvesting, the joint motion and muscle that potentially face MSD risk were compiled and illustrated in Fig. 6. It can be confirmed that the harvesters face higher MSD risk when performing back flexion and back rotation. The muscles responsible for these two motions are rectus abdominis, and the iliocostalis and the longissimus, respectively.

## Discussions

This study created a musculoskeletal model to investigate the harvesting motion. This model was adopted from a recent model by *Erica *et al*.*^18^, which was the first validated lifting model in OpenSim. The lifting motion requires the model to frequently flex the trunk and lift the object. Hence, the back muscles of the *Erica *et al*.* model were ensured to be strong enough to perform the lifting motion. This advantage made it preferred for the harvesting model because many qualitative studies reported that the FFB harvesters complained of low back pain^12,13,16^, indicating that strong back muscles were required during harvesting. Moreover, many current OpenSim models assume the trunk as one rigid body, such as the full-body models by Rajagopal et al.^40^ and Hamner et al*.*^41^, which is not true in reality and may not fully represent the dynamic behavior of the trunk. The trunk in the Erica et al*.* model was made of several rigid body segments, including each lumbar vertebrae (L1–L5), pelvis and torso^18^. This trunk structure is considered more accurate because the trunk of a real human is a chain of interconnected vertebrae, pelvis and scapula^1^. On the other hand, the biceps and triceps of the simulated harvester were added from another latest validated model by Daniel et al*.*^20^, which performed pushing and pulling tasks at different elevation angles. These muscles were added because the harvesting motion involves pulling motion, similar to the pulling task.

This study demonstrated a novel approach to investigate the worker's behavior in harvesting oil palm FFB. Wearable IMU allows the motion to be captured in the oil palm plantation without sophisticated setup and calibration. It is also proven to be cheaper, lighter and does not suffer from marker occlusion, making it an ideal alternative for outdoor applications^42^. The comparison between simulation and experimental results shows good to excellent correlations. This suggests that OpenSim can be a valid tool to determine the kinematic behavior of the FFB harvester's upper extremity.

Although not directly comparable, the R_θ_ and RMSE_θ_ reported in this study were close to those reported in other studies^43,44^. Rodriogo Perez et al.^43^ investigated simple shoulder, elbow and wrist joint motions using IMU sensors and validated them with MoCap. The shoulder AA (R = 0.718) showed the lowest correlation when compared to shoulder FE (R = 0.994) and shoulder rotation (R = 0.995). This finding is consistent with the current study, suggesting that it is difficult to obtain a higher correlation result for shoulder AA, even for simple motions. The authors then investigated the motions when the subjects served water from a jar. In this case, the shoulder rotation (R = 0.853) showed the lowest correlation when compared to shoulder FE (R = 0.996) and shoulder AA (R = 0.908)^43^. Jim Richards also pointed out that many papers focused on sagittal plane joint angles rather than the coronal and transverse planes^33^. These results support our findings, which show that shoulder FE has the highest correlation compared to other shoulder motions.

In another study^44^, Brice Bouvier et al*.* investigated simple elbow FE motions using IMU sensors. They validated their results against MoCap data and found RMSE value of 24°. A recent systematic review demonstrated that the validity of joint motion decreases when the level of complexity of the motion increases^36^. This implies that if the R_θ_ and RMSE_θ_ are similar or better than those studies with simple motions, then it can be deduced that the findings are acceptable. Since the RMSE_θ_ of elbow FE reported in this study (Dominant: RMSE_θ_ = 11.87°; Non-dominant: RMSE_θ_ = 20.22°) are less than the RMSE reported in^44^, therefore the results are satisfactory.

This study has also provided a unique mean to investigate the muscle activation of the oil palm FFB harvesting motion using musculoskeletal model. The muscle activations were validated with sEMG, which is the standard used in human motion analysis^30,45^. Other validation methods such as comparison with similar studies are impossible because very limited studies investigated the muscle behavior of FFB harvesters with EMG^11^. From Table 6, the normalized activations of the back muscles (longissimus and multifidus) showed a high correlation in R_MA_ and a good correlation in MAE_MA_ when F_RA_ were 30 N and 50 N. These findings concur with previous studies in lifting tasks^18,46^ and daily living activities^46^, which obtained high similarity results for the back muscles between the OpenSim muscle activations and EMG.

For the arm muscles, the triceps demonstrated a high correlation in R_MA_ and a good correlation in MAE_MA_. The biceps demonstrated a high correlation in R_MA_ and a poor correlation in MAE_MA_ when F_RA_ was 30 N. Three possible reasons may explain the poor correlation of biceps. Firstly, higher speed can result in higher deviations than lower speed^47^. The harvesters must exert alarge force at a very high speed to harvest the fruit^48^. Since the biceps is the primary muscle to perform this motion^11,49^, it is reasonable that the biceps showed higher MAE_MA_ values when compared to other muscles. Secondly, Roberto Bortoletto et al*.* proved that different F_RA_ could affect the simulated muscle activation in OpenSim^50^. From Table 6, it can be observed that the multifidus and longissimus showed the best correlation of R_MA_ and MAE_MA_ when the F_RA_ were 30 N and 50 N, respectively. The biceps showed the highest R_MA_ when F_RA_ was 30 N, whereas it showed the lowest MAE_MA_ when F_RA_ was 50 N. The triceps showed the highest R_MA_ when F_RA_ was 50 N, whereas it showed the lowest MAE_MA_ when F_RA_ was 30 N. These findings are valuable and important because currently there is no recommended value of F_RA_ for harvesting activity in OpenSim. Thirdly, the muscle architectural parameters of the simulated harvester may not represent the muscle architectural parameters of the real harvesters.

The potential risk of MSD that harvesters face during oil palm FFB harvesting were discussed based on the joint angle, muscle activation, muscle force and joint moment obtained with OpenSim. It was found that the back flexion and back rotation were the stressful joint motions during harvesting. Repetitive joint motion may lead to degenerative osteoarthritis^51^. During harvesting, the back muscles (longissimus, iliocostalis and rectus abdominis) were actively used. It was reported in^51^ that the overuse of muscle might cause myalgia. These findings are consistent with many qualitative studies on harvesters. Through questionnaires and direct interviews, many harvesters reported discomforts on their lower back^12,13,16,52^. Studies that used the direct observation assessment method found that the harvesters were facing a prevalence of MSD on their backs^16,49,52^.

Many qualitative studies reported that the harvesters suffered from low back pain. A few possible reasons can explain this issue. First, it was found that after a long duration of repeated back flexion, the stiffness of the spine decreased. The deformation of the intervertebral disc and the stretching of the ligaments in the spine generated a change in the loading pattern, leading to low back pain^53^. Second, during the flexions of the back and shoulder, the moment arms of the body segments increase, causing the joint moment and compensatory tension in the back muscles to increase. It is due to the extremely small moment arms of the back muscles; hence a large force is required to counteract the joint moment^53^. Lastly, it was known that the passive moment (i.e., moment due to passive structures such as ligaments, fascia and cartilage) increases exponentially when a joint is approaching the ROM of flexion^1^. A large increase of passive moment might also lead to low back pain in the harvesters. All these evidence supported our findings, which proved that the back flexion was the stressful joint motion during harvesting.

This study has several limitations. The muscle architectural parameters of the musculoskeletal model, such as the force–length relationship of muscle and tendon, may not represent the muscle architectural parameters of the harvesters^30,37,47^. Moreover, the assumption that the harvesting force was 303.5 N may not represent the actual harvesting force for each harvester. The harvesting force is also influenced by factors such as the frond maturity, cutting angle, speed of cutting and the sharpness of the cutting edge of the harvesting tools^54^. It is suggested that a mock harvesting environment can be prepared indoors. A more controlled environment will allow the other measurements such as MoCap and force sensor to improve the accuracy of the results. It has to bear in mind that although all these limitations limit the extent of interpretation, they do not invalidate the results^1^. The discrepancies between OpenSim and sEMG might be caused by the limitation of sEMG. There might be crosstalks and movement artefacts from neighbouring muscles^55,56^, especially during fast motions^30^. The small recording region of the sEMG electrodes on a particular muscle may not represent the whole muscle's activation^30^. The fine-wire EMG would be a more direct and accurate approach to validate the simulation results. However, it is invasive and is not practical for field study^30^.

To the best of our knowledge, it is the first study investigating FFB harvesting with musculoskeletal modelling and simulation. The joint angles and muscle activations were validated with field measurements to confirm the accuracy of the simulation results. Additionally, the computed findings provide additional biomechanical parameters that are impossible to obtain through any direct measurement or qualitative assessment. Consequently, these findings are beneficial to the designers of the wearable exoskeleton to identify the exact muscles and joints to improve the harvester’s strength during harvesting. This study also provides detailed insights to researchers in different areas dealing with computational biomechanical modelling, postural optimization and ergonomic assessments.

## Conclusion

This study created and evaluated an upper extremity musculoskeletal model for FFB harvesting. The joint angles were validated against the commercial IMU-based motion capture software, whereas the muscle activations were benchmarked against the EMG data. The findings concur with previous qualitative studies that suggested the harvesters are exposed to the ergonomic risk of MSD, particularly the back flexion and back rotation. The muscles actively used during harvesting were the longissimus, iliocostalis and rectus abdominis. These findings can provide essential information for future studies that focus on the ergonomics of the harvesting activity and the design and development of a wearable device to assist the harvester.
